# Draft Genome Sequence of *Ruminococcus* sp. nov. B05, Isolated from Human Feces

**DOI:** 10.1128/MRA.01458-18

**Published:** 2019-01-10

**Authors:** Chien-Hsun Huang, Jong-Shian Liou, Chun-Lin Wang, Lina Huang

**Affiliations:** a Bioresource Collection and Research Center, Food Industry Research and Development Institute, Hsinchu, Taiwan, Republic of China; University of California, Riverside

## Abstract

Ruminococcus sp. nov. B05 was isolated from a fecal sample of a 34-year-old adult male in Taiwan, Republic of China. The genome assembly comprised 3,576,560 bp, with a 38.71% G+C content.

## ANNOUNCEMENT

During a study aimed at isolating novel bacterial species found in the human gut, we isolated Ruminococcus sp. nov. B05 from a fecal sample of a 34-year-old adult male in Taiwan, Republic of China, using the culturomics approach (1–3). Based on the sequencing of the complete 16S rRNA gene, strain B05 was found to exhibit 95.19% sequence identity with Ruminococcus lactaris ATCC 29176^T^ (GenBank accession number GCA_000155205), which indicates that this isolate may represent a new species. In addition, the average nucleotide identity (ANI) and digital DDH (dDDH) values between strains B05 and ATCC 29176^T^ were 74.48% and 24.5%, respectively, which were clearly higher than the generally accepted cutoff thresholds of 95% to 96% and 70% for delineation of prokaryotic species (4, 5), and this thus confirms that strain B05 represents a novel species.

Ruminococcus sp. nov. B05 is a strictly anaerobic bacterium. It grows on yeast extract, casitone, fatty acid, and glucose (YCFAG) agar (6) in 48 h or less at 37°C under anaerobic conditions. A loop of cells was taken from the plate, and genomic DNA was extracted with an EasyPrep HY genomic DNA extraction kit (Biotools Co. Ltd., Taiwan) following the manufacturer’s protocol. Libraries were constructed with approximately 1 μg of genomic DNA with the NEBNext DNA library prep master mix set (NEB, Inc.), followed by paired-end (2 × 150-bp) sequencing on an Illumina HiSeq 4000 instrument at Beijing Novogene Bioinformatics Technology Co., Ltd. (Beijing, China). Sequencing the B05 strain generated about 1.4 Gbp paired-end raw reads. The low-quality paired-end sequences and adapter sequences were removed with an in-house program written by Novogene Bioinformatics Technology Co., Ltd. After adapter filtering and quality trimming, *de novo* assembly of the approximately 1.3 Gbp clean reads was performed with SOAPdenovo software version 2.04 under the default settings (7), which resulted in 70 scaffolds with an *N*_50_ value of 119,393 bp. The draft genome contains 3,576,560 bp, with a GC content of 38.71%, and it was annotated with the NCBI Prokaryotic Genome Automatic Annotation Pipeline (PGAAP) to obtain 3,405 predicted genes, 11 rRNAs, 60 tRNAs, and 4 noncoding RNAs (8). Annotation of the protein-coding genes with Rapid Annotations using Subsystems Technology (RAST) version 2.01 (9) predicted 3,434 protein-coding genes, 886 of which had functional categories of SEED subsystems, and analysis with antiSMASH version 4.2 (10) predicted 11 biosynthetic gene clusters, which include biosynthetic gene clusters for exopolysaccharides, pradimicins, polysaccharides, equibactin, and emulsan.

### Data availability.

This whole-genome shotgun project has been deposited at DDBJ/ENA/GenBank under the accession number RBZR00000000 (BioProject number PRJNA494619). The version described in this paper is the first version.
